# Labor division in collaborative visual search: a review

**DOI:** 10.1007/s00426-022-01767-8

**Published:** 2022-11-15

**Authors:** Basil Wahn, Laura Schmitz

**Affiliations:** 1 Institute of Educational Research, Ruhr-Universität Bochum, Bochum, Germany; 2 Department of Neurology, University Medical Center Hamburg-Eppendorf, Hamburg, Germany; 3 Department of Neurophysiology and Pathophysiology, University Medical Center Hamburg-Eppendorf, Hamburg, Germany

## Abstract

When looking for a certain object or person, individuals often engage in collaborative visual search, i.e., they search together by coordinating their behavior. For instance, when parents are looking for their child on a busy playground, they might search collaboratively by dividing the search area. This type of labor division in collaborative visual search could be beneficial not only in daily life, but also in professional life (e.g., at airport security screening, lifeguarding, or diagnostic radiology). To better understand the mechanisms underlying this type of collaborative behavior, as well as its benefits and costs, researchers have studied visual search scenarios in the laboratory. The aim of this review article is to provide a brief overview of the results of these studies. Are individuals faster if they search together compared to alone? And if so, should they simply search in parallel, or will they benefit from agreeing on a specific labor division? How should they divide the search space, and how to communicate this division? Should a consensus be reached (target present or absent?) before ending the search? We address these and further key questions, focusing on the aspect of labor division. In conclusion, we integrate the reviewed findings into an applied context, point out which questions still remain, and put forward suggestions for future research. We hope that this review can serve not only as a theoretical foundation for basic research but also as a practical inspiration for applied research and development.

## Introduction

When, in daily life, people are looking for a certain object or person, they often perform this task collaboratively by coordinating their behavior, and thereby facilitate the visual search (e.g., Malcolmson et al., 2007). For example, when parents are looking for their child on a large and busy playground, they might coordinate their search such that one of them looks around the left part of the area while the partner pays attention to the right. By doing so, they hope to spot their child faster than if they searched the ground independently (i.e., without coordinating). This type of labor division in collaborative visual search could be beneficial not only in daily life, but also in the professional sector, e.g., during lifeguarding at the beach, baggage screening at the airport (Enright & McCarley, 2019; Forlines et al., 2006; Malcolmson et al., 2007), or radiology at the hospital (also see Mitroff et al., 2015). Just like parents at the playground, professionals could divide up the search space (e.g., lifeguards divide the coastline, whereas doctors divide the X-ray image) to improve the efficiency of this crucial, and often time-critical, decision-making task.

To better understand the ways in which individuals manage to “coordinate cognition” (see Brennan et al., 2008) in these and similar scenarios, researchers have recently started studying collaborative visual search in the laboratory. The aim of this review article is to give a brief overview of the results of previous studies and to identify open questions that currently remain. Since the review specifically targets the aspect of labor division, it explicitly does not incorporate all published studies on collaborative visual search but only those which are informative with regard to labor division (15 articles in total (published between 2006 and 2021),^1^ three of which have been written by the first author of this paper). The review is structured into several interconnected research questions that build on each other. Before addressing each of these questions in turn, we will first introduce the experimental setup and task typically used to study collaborative visual search in the laboratory. We conclude this review with a summary of the key findings, possible applications thereof, and suggestions for future research.

## Collaborative visual search in the laboratory

**Fig. 1:**
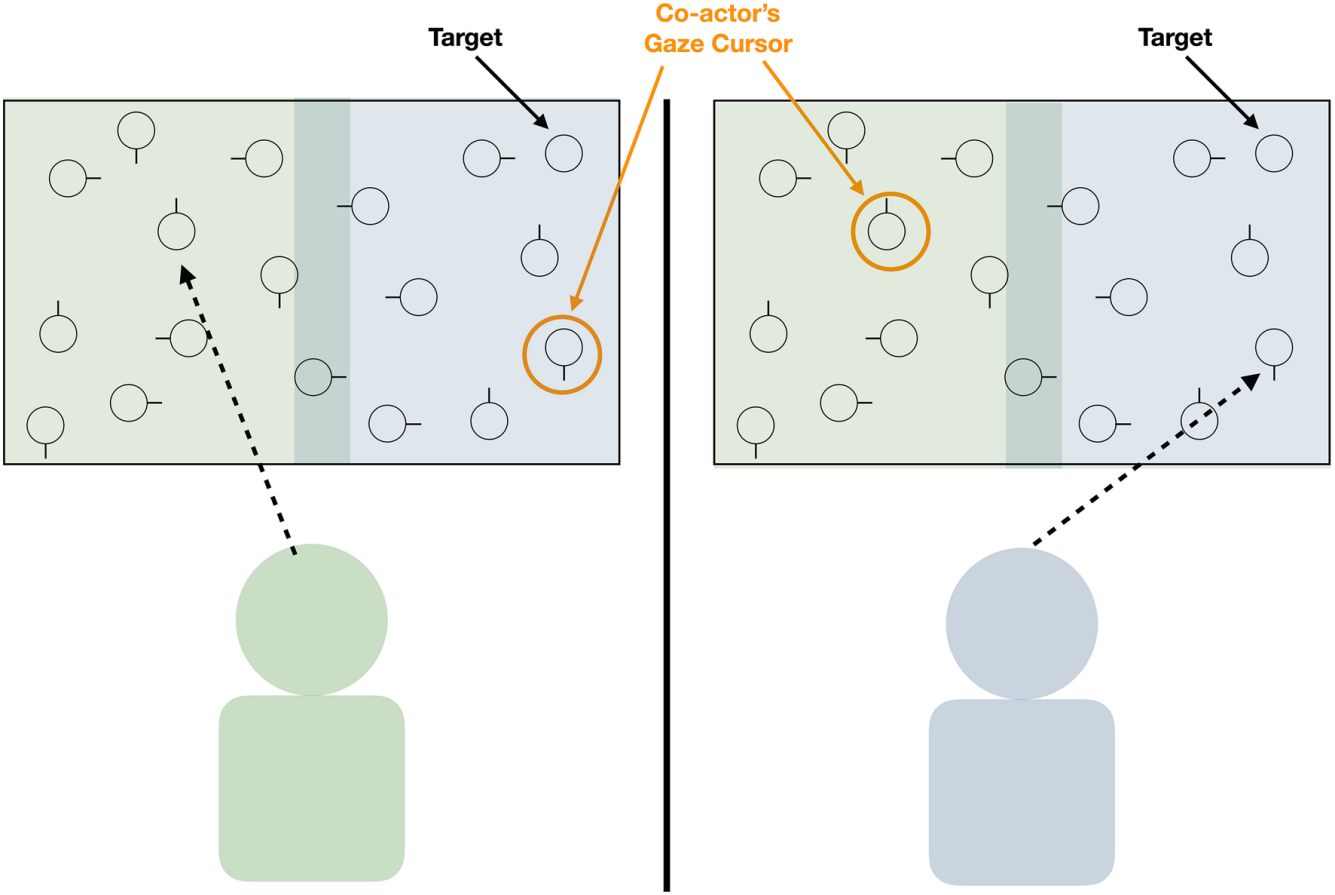
Exemplary setup for a collaborative visual search experiment. Two participants are seated side by side in front of two computer screens. The screens show a number of distractors (here: circles with antennas) and one target (here: a circle without antenna); the target is typically hard to detect among the distractors. Note that both screens show the same image. Participants are instructed to search for the target and decide whether it is present or absent. While searching, each participant is informed about the other’s current search location via a cursor (depicted in orange) which represents the other’s viewing direction (captured via eye tracking). In such experiments, participants often implement a form of labor division, e.g., by dividing the search space into a left (green participant) and a right (blue participant) area. Figure adapted from Wahn et al. (2018b) (colour figure online)

In a typical collaborative visual search experiment (e.g., see Wahn et al., 2020a), two participants are seated side by side in front of the same (or two identical) computer screen(s) and are instructed to search for a target among distractors displayed on the screen. For example, the target could be a circle and the distractors could be circles with small antennas (see Fig. 1). Target and distractors are located randomly across the screen, with the total number of items ranging, across different studies, between approximately 10 and 120. Importantly, the target is typically present only in half of all experimental trials (but see “Is a consensus decision necessary?”). The two participants are instructed to respond as fast and as accurately as possible (i.e., to decide, in each trial, whether the target is present or not). For example, participants might use a computer mouse to indicate whether the target is present (right click) or absent (left click). Usually, participants are also instructed to collaborate with each other. That is, they are told to solve the task together by coordinating their behavior. Importantly though, they are not told explicitly to divide the labor, but they are free to solve the task in their own way. Thus, if participants decide to divide the labor (as they often do), they do so spontaneously, without having been instructed by the experimenter. The search ends once one of the two participants gives a response or once both participants reach a consensus decision (see “Is a consensus decision necessary?”).

To determine whether searching together (collaborative search) is actually more efficient compared to searching alone (solitary search), studies typically include a (within- or between-subjects) baseline condition in which participants perform the same search task as described above, but individually. If search times for the collaborative search are shorter than for the solitary search, one can conclude that individuals benefit from searching together, i.e., they achieve what is often called a “group benefit” (e.g., Bahrami et al., 2010; Wahn et al., 2018b). That is, the term “group benefit” is used here to indicate that a group (note that, for the current purpose, we define a group as consisting of two or more people) searches faster than the group members search individually. Notably, previous studies have computed the group benefit in slightly different ways. Specifically, some studies have tested whether the group searches faster than its slower (or faster) group member searches individually (Brennan et al., 2015b; Wahn et al., 2018a, 2018b, 2020a, 2020b). Other studies, using between-subject-designs, have compared the average search time for groups with the average search time for individuals (Brennan et al., 2008; Siirtola et al., 2019).

Of course, what matters in a search is not only the time needed to complete it, but also the accuracy with which it is completed. After all, there is no benefit in completing a search really fast but making lots of errors (either by missing the target or by producing a false alarm). Thus, studies typically also measure *search accuracy* to test whether participants perform the task accurately. In most studies, accuracies are very high (> 90% accuracy) and accuracy differences between solitary search and collaborative search are negligible. For this reason, analyses focus mostly on search time as a measure of efficiency; we will, therefore, adopt the same focus here. This also means that, in the following, the term “(search) performance” always refers to search times, where a “better performance” or “performance gain” indicates shorter search times.

Studies differ not only in how they compare solitary with collaborative performance (as discussed above), but also with respect to whether, in the collaborative condition, participants (1) are allowed to exchange information (e.g., by talking to each other; see  “Which information is exchanged, and how?”), (2) need to reach a consensus decision (see “Is a consensus decision necessary?”), and (3) work in teams of two, three or four (see “How does increased group size affect labor division?”). Moreover, different studies report different labor division strategies (see  “How to divide the search space?”).

In the next section, we will first ask whether collaborating (by means of labor division) is actually beneficial—and what happens if group members do not collaborate. We will then ask what makes a labor division efficient, and describe which measures researchers have used to capture and quantify the efficiency of labor divisions.

## Is collaboration beneficial? And what makes a labor division efficient?

Before analyzing labor divisions in collaborative visual search, it is instructive to first ask what happens if two individuals (henceforth referred to as “co-actors”) search together, i.e., at the same time, yet without implementing any labor division. Would this type of parallel search still be faster than if individuals searched on their own? Going back to the example from the introduction: if two parents searched independently for their child (i.e., both looking around the playground yet without dividing the search space), would they still spot the child faster than if only one of them searched alone? To address this question, Wahn et al. (2018a) compared solitary search performance (i.e., participants performing the search task alone) with parallel search performance (i.e., two participants searching in parallel without any means of communication, and hence coordination). Results showed that, on average, the dyads searched faster than the individuals, indicating that searching together is indeed beneficial (Wahn et al., 2018a). Thus, a group benefit can be achieved even if co-actors act independently and without a labor division.

How does such a group benefit in parallel search come about? To determine the underlying mechanism, Wahn et al. (2018a) simulated participants’ dyadic performance using a race model (e.g., Colonius & Diederich, 2006; Gondan & Minakata, 2016; Miller, 1982). The race model operates on a trial-by-trial basis. It assumes that, during the parallel search, the target is always spotted first by that co-actor who, during the individual search, performed faster (compared to the other co-actor) on that particular trial. Indeed, this simulation closely approximates the actual dyadic performance that the researchers had observed in their behavioral study, indicating that the group benefit in parallel search can be ascribed to an underlying race model process.

However, as mentioned above, participants—if given the chance—most often do not search in parallel but search collaboratively by dividing the search space. How do researchers determine whether participants searched collaboratively, and how they divided the search space? In many studies, researchers asked participants after the experiment whether they had used any form of labor division strategy, and if so, which one (e.g., Malcolmson et al., 2007; Siirtola et al., 2019). While such subjective reports provide some first valuable insight into participants’ search behavior (and their awareness thereof), objective measures are needed to assess how efficiently the respective strategy actually functioned. For this purpose, researchers measured, via eye tracking technology, to what extent co-actors’ searched spaces overlapped (e.g., Brennan et al., 2008; Niehorster et al., 2019; Wahn et al., 2020a). A small or no overlap indicates that co-actors divided the search space efficiently (i.e., did not search the same space redundantly), whereas a large overlap indicates that the labor division was not implemented properly, as co-actors searched a large amount of space redundantly. The search overlap thus provides an objective measure of search efficiency.

Apart from looking at subjective reports and efficiency measures, participants’ labor division can also be quantified by measuring the performance gain in collaborative compared to solitary search. To do so, researchers have introduced a measure called “collaborative benefit” (Brennan & Enns, 2015b). This measure goes beyond the above-mentioned “group benefit” (i.e., faster performance when performing together compared to alone) by determining why groups searched faster than individuals. Specifically, if you observe a group benefit, the question is whether this benefit emerged because co-actors searched in parallel without using a labor division (see first paragraph of this section) or because co-actors actually collaborated and divided the labor. To determine the underlying cause for the group benefit observed in their study, Brennan and Enns (2015b) used a race model to simulate parallel search performance (just as Wahn et al., 2018a; see above). They then checked whether the empirical performance level surpassed the simulated one. If so, this performance difference quantifies the so-called “collaborative benefit”, i.e., the benefit that came about due to collaborative, rather than merely parallel, search behavior (for a recent review of this and other measures to quantify group benefits, see Wahn et al., 2018b). The size of the collaborative benefit presumably also reflects the quality of the labor division.

Finally, one should note that collaborating via labor divisions also comes with certain cognitive costs. On the one hand, costs occur when co-actors coordinate *how to* divide the labor. In particular, previous research looking at coordination in a visual tracking task has shown that cognitive costs, as indexed by pupil size, are higher when two co-actors need to coordinate a labor division compared to when this division is already predetermined (Wahn et al., 2021). Note that if a division is predetermined, however, this division cannot be adjusted to co-actors’ individual capabilities (e.g., such that the person who searches faster is assigned a larger proportion of the search space; see “How to divide the search space?”). On the other hand, costs occur when co-actors monitor each other’s behavior. In particular, previous research has shown, via workload analysis (Houpt et al., 2014), that individual capacity is reduced when co-actors keep an eye on each other’s current search location (Yamani et al., 2017). Individual capacity is reduced because co-actors need to perform two visual tasks at the same time (searching for the target and monitoring the co-actor’s search location), which creates dual-task interference (Yamani et al., 2017). The latter aspect is further discussed below.

## Which information is exchanged, and how?

To decide whether and how to divide the search space, co-actors need some way of exchanging information with each other. In principle, a labor division could be implemented accidentally without any information exchange (e.g., if co-actor A decides to search the left side of the screen and hopes that co-actor B searches the right, while co-actor B acts the other way around), yet this would depend in large part on coincidence rather than on strategic implementation. The most obvious way of exchanging information before or during a visual search is by talking to each other (see Forlines et al., 2006). For instance, one parent could say “I will search the left part of the playground around the slide.” and the other parent could agree “Okay, then I will search the right part around the swings”. Testing this everyday intuition in the laboratory, Malcolmson et al. (2007) investigated whether allowing co-actors to communicate verbally would enable them to implement a labor division. As expected, the majority of dyads reported that they divided the search space into two areas (see “How to divide the search space?”). Importantly, the results also showed that by doing so, co-actors attained a group benefit (Malcolmson et al., 2007). Note that in this study, compared to most others, group benefit refers to a benefit in terms of search accuracy (rather than search time). That is, dyads who could communicate showed a greater sensitivity (d’) for detecting targets compared to “nominal dyads” (i.e., two participants who searched in parallel without any means of communication).

While coordinating a spatial division in advance (e.g., “I search around the slide, you search around the swings.”) is certainly beneficial, it might be even more efficient if a spatial division could be coordinated more flexibly and dynamically during the search. Addressing this possibility, Brennan et al. (2008) provided each co-actor with information about the other co-actor’s current gaze location by displaying gaze cursors on the screen (see Fig. 1). This way, each co-actor knew where the other was currently searching and they could flexibly adapt to each other. Brennan et al. then compared the search time in this condition where information was exchanged via gaze (“shared gaze”) with a condition where co-actors exchanged information verbally (“shared voice”). In addition, the researchers included a solitary search condition as a baseline. Results showed that both types of information exchange (via gaze and voice) enabled co-actors to divide the search space. Importantly, gaze information enabled dyads to complete the search faster than if they used verbal communication, effectively halving the search times relative to solitary search (for recent replications, see Niehorster et al., 2019; Wahn et al., 2020a). This suggests that dynamic gaze exchange allows for a more efficient labor coordination than verbal exchange.

Importantly, Brennan et al. (2008) also verified that search performance in the “shared gaze” condition was actually better than performance in a parallel search condition, where co-actors search independently without exchanging information (see “Is collaboration beneficial? And what makes a labor division efficient?”). This was done by creating nominal pairs (by pairing individuals from the solitary search condition) and then picking, on each trial, the faster individual’s search time. Search times from this “nominal condition” were longer than those from the collaborative “shared gaze” condition. Similar results were obtained by Niehorster et al. (2019) who compared the collaborative condition to what they called a “blind simulation”. Brennan et al.’s finding was again replicated, in a within-subject design, in a recent study by Wahn et al. (2020a), confirming that co-actors who received gaze information and divided the search space performed considerably faster than co-actors who merely searched in parallel. Another study (Brennan & Enns, 2015a) compared parallel search performance with performance in a “shared voice” condition, demonstrating that co-actors who could verbally communicate also searched faster than if they searched in parallel without verbal exchange. Yet this was only true if co-actors had visual access to each other (allowing for gestures as an additional information channel) and if they had a friendly relationship (Brennan & Enns, 2015a). Together, these studies show that search times are faster when co-actors are able to exchange information (e.g., via shared gaze) compared to when they perform an independent, parallel search without information exchange.

Notably, shared gaze during collaborative visual search comes not only with benefits, but also with certain costs. Specifically, if co-actors constantly see a moving gaze cursor on the screen (which represents the respective other’s current gaze location), this cursor might present a source of distraction because motion cues generally have a strong bottom-up saliency and automatically attract attention (Wolfe & Horowitz, 2017). To circumvent this problem, one could present gaze information in different, less distracting ways. This was done by Niehorster et al. (2019) who displayed a larger gaze area (rather than a small gaze cursor) to highlight co-actors' gaze locations to reduce the frequent jitter that occurs when the highlighted location changes all the time. However, participants reported that they still found this display moderately distracting. Zhang et al. (2017) introduced a possibly even subtler presentation (namely increased brightness of the co-actor’s gaze location) and found that this mode of presentation is preferred by participants and perceived as less intrusive than more explicit presentations such as the solid gaze cursor used by Brennan et al. (2008). These insights could be of applied importance when it comes to usability and user experience.

The potential dual-task interference between two visual tasks as described above (i.e., searching for the target and monitoring the co-actor’s gaze) could, however, be circumvented if gaze information was presented via a different sensory modality than vision. This would be beneficial because attentional capacity is larger if incoming information is distributed across sensory modalities, e.g., across the visual and the auditory modality (Alais et al., 2006; Arrighi et al., 2011; Wahn & König, 2016; for a review, see Wahn & König, 2017). Wahn et al. (2015) tested this hypothesis by providing co-actors with gaze information via the tactile or auditory modality. This was done by providing participants with tactile vibrations (via a vibrotactile belt) or auditory tones (via headphones). The spatial location of these vibrations/tones corresponded to the location of the co-actor’s current gaze location on the computer screen. The spatial correspondence was achieved by dividing the computer screen into 21 sections (not visible to participants) and assigning each section to a specific vibromotor on the belt or a specific tone. Motors and tones were spatially arranged in the same 21 sections as the computer screen (i.e., the belt had a corresponding layout and the tones were simulated such that they were perceived as originating from the corresponding spatial locations); see Wahn et al. (2015) for details.

Results from that study (Wahn et al., 2015) showed that for the search phase, providing gaze information via the auditory channel led to significantly faster search performance than providing the same information via the visual channel. The opposite was true for the consensus phase (i.e., the phase in which one participant has found the target and the other participant needs to confirm this choice by fixating the same location; see “Is a consensus decision necessary?”), where search performance was faster in the visual compared to the auditory condition. In the auditory condition, the faster search times during the search phase can be explained by the larger attentional capacity mentioned above (Wahn & König, 2017), which allowed co-actors to search for the target (visually) while at the same time monitoring each other’s gaze location (auditorily). In the visual condition, the faster search times during the consensus phase are likely due to the higher spatial precision of visual compared to auditory information (Körding et al., 2007; Rohe & Noppeney, 2015; Wahn et al., 2015), which made it easier for co-actors to realize if and where their partner had found the target.

Together, the studies discussed above suggest that information exchange during collaborative visual search is beneficial, yet only if the information is exchanged in ways that minimize distraction (e.g., by displaying information in non-intrusive formats or by distributing information across different sensory modalities).

## Is a consensus decision necessary?

While the above studies (Brennan et al., 2008; Niehorster et al., 2019; Wahn et al., 2020a) clearly show the benefits of information exchange during collaborative visual search, one should note that these studies did not include a consensus decision (but see Wahn et al., 2015). That is, the two co-actors in these studies did not have to agree on a decision (target present or absent?) but rather, the one to respond first was the one to decide. Other studies (Messmer et al., 2017; Neider et al., 2010; Wahn et al., 2015; Yamani et al., 2017) added a so-called “consensus phase” to the search task, requiring co-actors to both fixate the target at the same time before being able to end the search. Note that in these studies, the target was present in all trials. The results of these studies showed that co-actors benefited from shared gaze specifically during the consensus phase, yet not so much during the preceding search phase. This is likely because once one of the two co-actors had found the target, the other would quickly realize (thanks to the gaze information) that her partner had stopped searching and could then fixate the same location. During the search phase, however, co-actors now tried to search for the target while at the same time monitoring their co-actor’s gaze movements (did he/she stop moving?) to be able to react quickly if the other had found the target. This monitoring behavior presumably created dual-task interference (Yamani et al., 2017), which increased individual search time and thereby overrode the benefits of shared gaze as originally observed by Brennan et al. (2008).

## How to divide the search space?

As described above, studies on collaborative visual search consistently report that participants generally aim to divide the search space on the computer screen. However, co-actors do not seem to have a clear preference for a vertical (left–right) or horizontal (up–down) division of the screen. While the most frequently reported strategy is a left–right division (e.g., Malcolmson et al., 2007; Wahn et al., 2015), where one co-actor searches the left part of the screen and the other co-actor searches the right, an up–down division has also been reported (Brennan et al., 2008; Siirtola et al., 2019). Regardless of the type of division co-actors choose, this division is typically implemented quickly within a few experimental trials (Siirtola et al., 2019).

Do co-actors split the search space evenly? Several studies report an equal division, where co-actors search roughly an equal amount of screen space (Brennan et al., 2008; Siirtola et al., 2019; Wahn et al., 2020a). While it may appear reasonable and fair to split the labor in half, this approach is only optimal if the co-actors’ individual search abilities are also equal. Here, the term “optimal” indicates that two co-actors maximize their potential group benefit by reducing their search time as much as possible compared to solitary search.

Indeed, if two co-actors’ individual abilities are very similar and they divide the labor in half, they tend to gain a larger group benefit compared to two co-actors’ whose individual abilities greatly diverge (Wahn et al., 2020a). In the latter case, an optimal strategy would be to divide the labor relative to the co-actors’ individual abilities. For example, if one co-actor searches twice as fast as the other, the former one should take over two-thirds of the search space. Still, most co-actors prefer an equal division of labor. We will return to this point in “Summary and applications”.

## How does increased group size affect labor division?

In the majority of studies, *two* participants perform the collaborative search task together. While there are costs to coordinating labor in dyads (see “Is collaboration beneficial? And what makes a labor division efficient?” and “Which information is exchanged, and how?”), such coordination costs increase with increased group size. To test whether the benefits of coordination still outweigh its costs even in larger groups, two recent studies (Siirtola et al., 2019; Wahn et al., 2020a) investigated whether the benefits of shared gaze for dyads (Brennan et al., 2008; see “Which information is exchanged, and how?”) scale up to triads. While both studies found that triads outperformed dyads, they also found that the triads’ performance gain was not as large as expected if coordination costs had been the same as during dyadic search. The additional coordination costs during triadic search are clearly reflected in an increase of search overlap (see “How to divide the search space?”) as well as in participants’ subjective reports about less structured labor divisions (Wahn et al., 2020a).

The latter finding suggests that the benefits of coordination in visual search (i.e., a decrease in search times) may be offset by its costs if the optimal group size is exceeded (i.e., the gain in search time does not correspond to the number of additional team members). In collaborative visual search tasks in the laboratory, the optimal group size seems to be two. However, the optimal size very likely depends on the specific task and task conditions, and thus might differ between laboratory and applied contexts. For example, one study (Forlines et al., 2006) used a baggage screening task where co-actors searched simulated X-ray images for prohibited items and found that teams of four co-actors committed fewer errors and performed (descriptively) faster than teams of two, yet only if the target was present. Combining these results by Forlines et al. with the above-mentioned results by Wahn et al. (2020a), it seems that adding two individuals to a group of two is more beneficial than adding just one individual. The reason could be that it is less intuitive how to divide a rectangular search space into three compared to four equal parts. In the latter case, one would intuitively divide the space into quadrants, whereas in the former case, the spatial division is not as straight-forward (e.g., one could have three horizontal sections or three vertical sections). Thus, the ease of task division might be one factor that determines which group sizes are optimal for a given task (also see Wahn et al., 2020a). Future research is needed to determine further potential factors.

## Summary and applications

In this review, we aimed to provide a brief overview of recent research on collaborative visual search, focusing on the aspect of labor division. As an intuitive example of collaborative visual search, we imagine two parents looking for their child on a busy playground. Can they spot their child faster if they search together compared to alone? And should they simply search in parallel, or will they benefit from agreeing on a specific labor division (e.g., “I search around the slide, you search around the swings.”)? How should they divide the search space, and how to communicate this division? If one parent thinks they might have spotted the child, would it be useful to first check back with the partner (“Is that really him in the orange jacket over there, or did he wear the green jacket today?”) and reach a consensus? Would it be helpful if a friend helped them looking? All of these questions have been addressed above, drawing on empirical evidence from collaborative visual search (not on the playground but) in the laboratory.

Together, the results indicate that (1) two co-actors achieve a “group benefit” if they search together rather than alone (meaning that the collaborative search is faster than the solitary search), even if they search in parallel (i.e., independently) without implementing a labor division (Wahn et al., 2018a); yet (2) the size of that group benefit is considerably larger if co-actors actively collaborate and divide the labor than when they search independently (Wahn et al., 2020a); (3) labor division can be quantified by asking participants for subjective reports (Wahn et al., 2015), by measuring the search overlap (Brennan et al., 2008; Niehorster et al., 2019; Wahn et al., 2020a), and by computing a so-called collaborative benefit (Brennan & Enns, 2015b); (4) exchanging information via voice or gaze is sufficient to implement a labor division (Brennan et al., 2008); (5) a mandatory consensus decision can create negative interference effects (Messmer et al., 2017; Neider et al., 2010; Yamani et al., 2017) which, however, can be circumvented by distributing task demands across different sensory modalities (Wahn et al., 2015); (6) co-actors divide the search space evenly (either using a left–right or an up–down division of space; Malcolmson et al., 2007), regardless of individual search abilities (Wahn et al., 2020a), and the division is implemented quickly (Siirtola et al., 2019); (7) the benefits of increasing the group size beyond two can be outweighed by the additional costs of coordination (Siirtola et al., 2019; Wahn et al., 2020a) yet optimal group size seems to depend on the ease of task division (Forlines et al., 2006).

In the following, we will draw initial conclusions from the above findings, especially with regard to applied contexts. As mentioned at the outset of this paper, collaborative visual search could potentially be applied in various professional fields, such as, for example, radiology, airport security screening (Enright & McCarley, 2019; Forlines et al., 2006), cytology, lifeguarding, and termite inspection (see Mitroff et al., 2015). Professionals in these fields typically face high attentional demands and often work under enormous time pressure. For example, when looking for a suspicious item in an X-ray image of a carry-on bag, a decision (target present or absent?) has to be taken within seconds. This of course increases the risk of costly errors. By performing a search task together with another person, individual attentional demands could be reduced and errors avoided, leading to an overall better performance. While it would not even be necessary that co-working individuals communicate and actively collaborate, this would of course be preferred, because performance gains are greater for collaborative compared to independent search (Wahn et al., 2020a). If a visual search is performed on a computer screen (such as at airport security), one could employ modern eye tracking technology to provide co-workers with information about each other’s search location via non-intrusive gaze markers (Brennan et al., 2008; Zhang et al., 2017). A less technologically costly way would be for co-workers to exchange information verbally. In order not to compromise search time, co-workers should not be required to reach a consensus decision as this might create dual-task interference (see Yamani et al., 2017, and below). However, a mandatory consensus could be introduced in particularly crucial or perceptually difficult cases (on joint perceptual decision-making with mandatory consensus, see Bahrami et al., 2010).

Another reason why collaborative visual search might be beneficial in professional contexts is that during real-life searches, the number of targets is often unknown (a screened carry-on bag could potentially contain two prohibited items, e.g., a container with fluid *and* a pair of scissors, or no items at all). On the one hand, it has been shown for solitary search that the risk of missing additional targets is very high once a first target has already been identified (e.g., Berbaum & Franken, 2011; Biggs, 2017; “satisfaction of search effect”). On the other hand, one should consider that target prevalence is typically low in real-world tasks, such as during baggage screening or X-ray analysis, where the occurrence of a knife or a tumor is quite rare (see Forlines et al., 2006; Wolfe et al., 2005). It has been shown for solitary search that if targets are rare, they are more easily missed (Wolfe et al., 2005). Crucially, the risk of missing additional targets (see Brennan & Enns, 2015b) or rare targets (see Forlines et al., 2006) could be effectively reduced by performing the search together instead of alone.

In sum, it seems worthwhile to consider whether, in certain professional settings, one could transform the standard solitary visual search into a collaborative visual search to decrease search time. Without doubt, adding additional workers is economically costly. Therefore, we suggest to find an appropriate cost–benefit ratio by choosing the optimal group size yet not exceeding it (Siirtola et al., 2019; Wahn et al., 2020a). This way, one could maximize the benefits of coordination without incurring too many costs.

## Future directions

In the final section of this paper, we will identify several open questions and put forward suggestions for future research. One open question is concerned with how to (best) divide a search space (see “How to divide the search space?”). As reported above, it seems that when two co-actors divide the search space on a computer screen, there is no clearly dominant strategy; some use a vertical (left–right) and others a horizontal (up–down) division. Regardless of which type of division they choose, most co-actors prefer an equal division of labor, even if this is not the most beneficial strategy (see “How to divide the search space?”). Why do most co-actors still prefer equality? An equal division may be preferred out of a sense of fairness, or ease of implementation, or because co-actors assume that their individual search abilities are equal and hence, an equal division should be optimal. Further research is needed to determine which of these reasons can best account for the observed preference.

Moreover, regarding the type of division (vertical or horizontal), it would be helpful to assess which type of division strategy is most efficient and whether the strategy co-actors choose depends on particular environmental factors. In the experimental setup typically used to study collaborative visual search (see Fig. 1, “Collaborative visual search in the laboratory”), two such factors might be the shape (i.e., width–height ratio) of the computer screen and the seating position of the two participants (i.e., whether they sit side by side or opposite of each other). A recent study (Wahn & Kingstone, 2020) has investigated these factors, yet not using a search task but a related visuospatial task (multiple object tracking; Luo et al., 2021; Meyerhoff et al., 2017) in a collaborative setting. The study found that the shape of the screen determined how co-actors divided the space: if the screen was higher than it was wide (portrait), most participants chose an up–down division, whereas if the width of the screen was greater than its height (landscape), there was no clear preference across participants. The seating position did not have an influence on participants’ division strategy. Future studies could test whether the same holds for collaborative visual search. Besides determining the factors that influence participants’ choice, one should also determine which division strategy is the most efficient. Once the influencing factors (which strategy is chosen under which conditions?) and the efficiency of the different strategies is known, one could strategically design the task environment in ways that promotes the most efficient strategy (also see Forlines et al., 2006). This insight could then be applied to professional contexts, e.g., when deciding on the shape of the computer screens to be installed at airport security screening stations.

As previous research has shown that information exchange between co-actors can facilitate coordination during visual search (see “Which information is exchanged and how?”), it is important that, in applied contexts, this information exchange is designed such that any distracting effects are minimized. In particular, previous studies have provided each co-actor with information about the other co-actor’s current gaze location by displaying gaze cursors on the screen (Brennan et al., 2008; Wahn et al., 2015). When co-actors received this information, they found the target faster than if they did not receive any information. However, providing gaze information on the screen can be distracting (see Niehorster et al., 2019; Zhang et al., 2017), as the additional visual input might create dual-task interference (Yamani et al., 2017). To circumvent this interference in applied contexts, one could provide the additional information via a modality other than vision, e.g., by providing tactile or auditory information (Wahn et al., 2015). Previous research in the field of human factors has already demonstrated the benefits of providing information via tactile displays (in addition to visual displays), e.g., in navigation tasks in driving or flight simulators (Nikolic et al., 1998; Sklar & Sarter, 1999; Van Erp & Van Veen, 2004).

Another aspect worthwhile to investigate is whether the interpersonal relationship between two co-actors affects the way they collaborate during visual search. Previous research showed that co-actors with higher social affiliation scores (reflecting the closeness of a relationship) tended to attain higher group benefits during collaborative visual search (Brennan & Enns, 2015b) and that co-actors who were friends outperformed co-actors who were strangers (Brennan & Enns, 2015a). Moreover, findings from a collaborative multiple object tracking task (see above; Wahn et al., 2020b) showed that co-actors’ empathic abilities (as measured by the Interpersonal Reactivity Index; Davis, 1980) were correlated with their collaborative performance. Future research could test whether a similar relationship holds for collaborative visual search, and whether there are further interpersonal/social factors that influence the quality of collaboration. Once these factors have been established, one could use this knowledge in the professional sector to develop team building measures that promote specific aspects of co-workers’ interpersonal relationship, and thereby improve their collaborative abilities which, in turn, should positively affect their performance.

Moreover, one could further investigate to what extent the individual co-actors’ level of search ability/expertise affects how well they perform together. What if one individual has a very poor search performance, while the other individual excels at the task? Previous research in a related task domain (i.e., visual contrast discrimination) has shown that two individuals achieve a group benefit only if they have nearly equal perceptual sensitivities (Bahrami et al., 2010; Bang et al., 2014), i.e., if their task performance levels are similar. This is likely because for two co-actors with very different sensitivity levels, the more sensitive individual will still feel obliged to consider the less sensitive individual’s contribution (“equality bias”), which will negatively impact the group’s performance (see Mahmoodi et al., 2015).

Besides looking at individual differences in perceptual ability, one could also consider differences in social status. Previous research on social gaze has shown that individuals’ looking behavior is affected by the social status (higher or lower rank) of an interaction partner (Gobel et al., 2015, 2018), suggesting that visual attention is guided not only by the physical environment but also by its social relevance. Thus, for the present context of collaborative visual search, one could investigate whether (and if so, how) collaboration in general, and division of labor specifically, is affected by co-actors’ social ranks.

Another direction for future research could be to record physiological measures (e.g., participants’ heart rate, skin conductance, cortisol level, pupil size, etc.) during collaborative visual search, to better understand the underlying cognitive processes. For example, a previous study (Brennan & Enns, 2015a) measured participants’ arousal (via skin conductance and heart rate) to test whether group benefits might (partially) be attributable to social facilitation effects (i.e., performance benefits due to the mere presence of another person; Belletier et al., 2019, but see Oliva et al., 2017). Based on the arousal measures, the researchers concluded that social facilitation by itself could not explain the observed group benefits, but rather, these benefits arose from co-actors’ collaborative efforts (for details, see Brennan & Enns, 2015a). A different study simultaneously measured two co-actors’ electrical brain activity via EEG hyperscanning (for reviews on hyperscanning in social neuroscience, see, e.g., Hamilton, 2021; Konvalinka & Roepstorff, 2012) and found that measures of inter-brain synchronization positively correlated with dyadic performance during collaborative visual search (Szymanski et al., 2017). Another interesting measurement to record would be pupil size (Mathôt, 2018) and Event-Related Potentials (Drew et al., 2009) as a proxy of mental effort. Previous research has shown that pupil size increases with the costs of coordinating during a visual tracking task (Wahn et al., 2021). Collecting these and other physiological measures could nicely complement the behavioral findings reported above.

To conclude, even though this last section shows that there are still many open questions to be addressed in the future, we have seen that the research reviewed above already sheds some substantial light on how people coordinate their behavior during collaborative visual search.

## Footnotes

1 These articles are marked with an asterisk in the list of references.
